# Emerging Anti-Microbial Resistance in Febrile Neutropenia: Is it high time to evaluate quality control measures?

**DOI:** 10.12669/pjms.36.6.2138

**Published:** 2020

**Authors:** Uzma Mahar, Nida Anwar, Naveena Fatima, Jawad Hassan, Tahir Shamsi

**Affiliations:** 1 Uzma Mahar, (FCPS Resident) Department of Hematology, National Institute of Blood Disease & Bone Marrow Transplantation, Karachi, Pakistan; 2 Nida Anwar, (FCPS), Department of Hematology, National Institute of Blood Disease & Bone Marrow Transplantation, Karachi, Pakistan; 3 Naveena Fatima, (Pharm -D) Department of Research and Development, National Institute of Blood Disease & Bone Marrow Transplantation, Karachi, Pakistan; 4 Jawad Hassan, (FCPS) Department of Hematology, National Institute of Blood Disease & Bone Marrow Transplantation, Karachi, Pakistan; 5 Tahir Shamsi, (FRCPath) Department of Hematology, National Institute of Blood Disease & Bone Marrow Transplantation, Karachi, Pakistan

**Keywords:** Anti-Microbial Resistance, Febrile Neutropenia, Quality Control

## Abstract

**Objective:**

We performed a prospective analysis at our center to find out the most common organisms causing bacterial infections to establish pattern of antibiotic resistance, in order to combat febrile neutropenia effectively in the terms of outcome as well as cost.

**Methods:**

A hospital based observational study was conducted at National Institute of Blood Diseases and bone marrow transplantation from January 2017 to December 2017. Patients presented with absolute neutrophil count (ANC) of less than 500/ml were enrolled. Data were analyzed by SPSS version 21.0. P value of <0.05 was considered statistically significant.

**Results:**

In this study, a total of 242 patients from various hematological disorders were enrolled and 403 bacterial isolates were obtained. The most frequent isolated gram-negative organisms were *Escherichia coli*, followed by *Klebsiella pneumoniae* and the most prevalent gram-positive organisms were *staphylococcus aureus* and *Enterococcus species*. The antimicrobial susceptibility testing revealed that most of the *Staphylococcus aureus* isolates were highly resistant to methicillin (p=0.002), whereas *Enterococcus species* were resistant to vancomycin (p=0.000).

**Conclusion:**

The choice of empirical antibiotic regimen should be based on local spectrum of bacteria and their regional susceptibility pattern to improve the survival and minimize hospital stay of patients.

## INTRODUCTION

Febrile neutropenia is a medical emergency and patients with hematological disorders do encounter it either as a part of disease process or secondary to chemotherapy and/or radiation.^1^ Around 80% of the neutropenic patients develop fever.^2^ However, because of inadequate cytokines release the signs of infection/inflammation can be masked and may result in increased mortality rates.^3^ So, early commencement of empirical intra-venous (IV) antibiotic has crucial role and delay in initiation of treatment is associated with higher death rates.^4^

Apart from chemotherapy, there are multiple other factors which are responsible for febrile neutropenia and its complications.^5^ One of the factor is age which plays a significant role as older individuals are more prone to become febrile after chemotherapy.^5^ Other risk factors proposed by multinational association for supportive care in cancer include: burden of illness; hypotension, chronic obstructive pulmonary disease; fungal infection; dehydration and outpatient status.^6^ A local study done on patients with solid malignancies found that male gender is at higher risk to develop febrile neutropenia post chemotherapy as compare to female gender.^7^ Another indegenous study where gender distribution was equal in the study groups reported slightly higher incidence of mortality in males.^8^ Apart from the risk factors, local anti-microbial resistant profile also plays a crucial role in the outcome of febrile neutropenia.^9^

In 1950s and early 1960s the most common organism isolated was *S. aureus* which was replaced by gram negative organisms later on. However, reemergence of gram positive organisms was again noted in 1980s.^10^ But in the last two decades, increased risk of multi drug resistant infections have been reported worldwide. Most prevalent multi drug resistant gram positive bacteria include methicillin resistant *Staphylococcus aureus* and vancomycin resistant *Enterococci* 3 and multi drug resistant gram negative bacteria are those which are resistant to at least three of the following antibiotic groups: i.e. anti pseudomonal penicillin, cephalosporins, carbapenems, aminoglycosides and fluoroquinolones.^3^,^11^

Increased use of broad spectrum antibiotics has resulted in resistant bacteria and different centers have different prevalence and trends of resistance. Therefore, it is important to check the prevalence of micro-organism and its sensitivity pattern to carefully design antibiotic regimens and review the institutional polices regarding the use of empirical antibiotic in patients of febrile neutropenia. Since the 1990’s, many studies have been conducted on prophylactic use of antibiotics to cope up with febrile neutropenia.^12^ However, on the other hand according to a study the prophylactic use of such antibiotics has resulted in resistant strains and thus it is emphasized that prophylactic use of antibiotics should be discouraged.^5^,^12^

With this background, we performed a prospective analysis at our center to find out the most common organisms causing bacterial infections to establish pattern of antibiotic resistance, in order to combat febrile neutropenia effectively in the terms of outcome as well as cost.

## METHODS

A hospital based observational study was conducted at National Institute of Blood Diseases and bone marrow transplantation from January 2017 to December 2017. This study was approved by Institutional Review Board .(Ref.NIBD/RD-173/05-2017) Patients presented with absolute neutrophil count (ANC) of less than 500/ml were enrolled and informed consent was taken. Patients presenting with absolute neutrophil count of less than 500/ml and with fever defined as a body temperature of ≥38.5 C. Patients who were on quinolone prophylaxis or with known hypersensitivity to any of the prescribed antibiotics in febrile neutropenia were excluded from the study. Blood cultures were processed using the BACTEC blood culture system. Organisms were identified according to routine bacteriological procedures. Antibiotic susceptibility testing was interpreted by disc diffusion method. Results were interpreted according to the Clinical and Laboratory Standards Institute’s criteria. Data was analyzed by SPSS version 21.0. P value of <0.05 was considered statistically significant. Data was described as frequencies and percentages and categorical variables were compared using the chi-square test or Fisher’s exact test for association.

## RESULTS

In our study, 403 bacterial isolates were collected from 242 patients suffering from various hematological disorders and specimens consisted of 157 urine, 83 peripheral blood, 61 Hickman line, 55 throat swabs and 34 pus cultures. The most prevalent organisms in present study were gram negative bacteria which consisted of 302 (74.9%) isolates however 101 (25%) isolates revealed growth of gram positive organisms. In gram negative, *Escherichia coli* was found to be more prevalent 121(40%) than *Pseudomonas aeruginosa* 85(28%), *Klebsiella pneumoniae* 82(27%), *Salmonella sp*. 11(4%) and *Proteus mirabilis* 3(1%) whereas in gram positive *Staphylococcus aureus* 56(55%) was more prevalent than *Enterococcus* Species 36(36%), β Hemolytic *Streptococcus* Group A 5(5%) and *Streptococcus epidermidis* 4(4%). Occurrence and association of gram negative and gram positive organisms in different specimens is shown in Table-I and II.

**Table-I:** Occurrence and association of gram negative organisms (n=302) in different specimens.

Specimen	Escherichia coli N(%)	Proteus mirabilis N(%)	Klebsiella pneumoniae N(%)	Pseudomonas aeruginosa N(%)	Salmonella sp. N(%)	P-value
Pus C/S, N=16	2(12)	1(6)	6(38)	7(44)	0	0.000
Urine C/S, N=129	79(61)	2(2)	19(15)	29(22)	0	
Hickman Line, N=47	16(34)	0	12(26)	19(40)	0	
Peripheral, N=60	16(27)	0	20(33)	14(23)	10(17)	
Throat swab C/S N=41	8(200	0	22(53)	11(27)	0	
Sputum for C/S N=05	0	0	2(25)	3(75)	0	
Wound Swab	0	0	0	0	0	
Nose Swab	0	0	0	0	0	
Bone marrow C/S N=04	0	0	1(25)	2(50)	1(25)	

**Table-II:** Occurrence and association of gram positive organisms (n=101) in different specimens.

Specimen	Streptococcus epidermidis N (%)	Staphylococcus aureus N (%)	Enterococcus Species N (%)	β Hemolytic Streptococcus Group A N (%)	P-value
Pus C/S, N=18	2(11)	15(83)	0	1(6)	0.000
Urine C/S, N=28	0	2(7)	26(93)	0	
Hickman Line N=14	0	8(57)	6(43)	0	
Peripheral, N=23	2(9)	16(70)	4(17)	1(4)	
Throat Swab for C/S, N=14	0	12(86)	0	2(14)	
Sputum for C/S, N=01	0	0	0	1(100)	
Wound Swab, N=01	0	1(100)	0	0	
Nose Swab, N=02	0	2(100)	0	0	
Bone Marrow C/S	0	0	0	0	

The sensitivity pattern and association of antibiotics to gram positive and gram negative organisms shown in Table-III & IV. Overall, piperacillin-tazobactam and meropenem resistance in gram negative organisms was found to be 20% and 17% respectively, while in gram positive organisms it was 71% and 69%.

**Table-III:** Association of resistance and sensitivity of antibiotics in isolates of gram positive organisms.

Antibiotics	Resistance /Sensitivity	Streptococcus epidermidis N (%)	Staphylococcus aureus N (%)	Enterococcus Species N (%)	β Hemolytic Streptococcus Group A N (%)	P-value
Ampicillin (n=100)	R (n=78)	0	53(53)	25(25)	0	0.000
	S (n=22)	4(4)	2(2)	11(11)	5(5)	
Amox-clav (n=100)	R (n=71)	2(2)	44(44)	25(25)	0	0.002
	S (n=29)	2(2)	11(11)	11(11)	5(5)	
Piperacillin- Tazobactam (n=100)	R (n=71)	2(2)	44(44)	25(25)	0	0.002
	S (n=29)	2(2)	11(11)	11(11)	5(5)	
Ceftriaxone (n=100)	R (n=78)	3(3)	44(44)	31(31)	0	0.000
	S (n=22)	1(1)	11(11)	5(5)	5(5)	
Vancomycin (n=96)	R (n=16)	0	2(2)	14(15)	0	0.000
	S (n=80)	4(4)	50(52)	21(22)	5(5)	
Meropenem (n=98)	R (n=68)	2(2)	41(41)	25(26)	0	0.004
	S (n=30)	2(2)	13(13)	10(10)	5(5)	
Amikacin (n=92)	R (n=44)	0	9(10)	35(38)	0	0.000
	S (n=49)	4(4)	44(48)	1(1)	0	
Ciprofloxacin (n=100)	R (n=77)	2(2)	42(42)	33(33)	0	0.000
	S (n=23)	2(2)	13(13)	3(3)	5(5)	
Fosfomycin (n=98)	R (n=17)	0	6(6.1)	11(11)	0	0.032
	S (n=81)	4(4)	49(50)	23(23)	5(5)	
Neomycin (n=19)	R (n=7)	0	7(37)	0	0	0.354
	S (n=12)	2(11)	9(48)	0	1(5)	

R=resistance, S= sensitivity

**Table-IV:** Association of resistance and sensitivity of antibiotics in isolates of gram negative organisms.

Antibiotics	Resistance /Sensitivity	Escherichia colis N(%)	Proteus mirabilis N(%)	Klebsiella pneumoniae N(%)	Pseudomonas aeruginosa N(%)	Salmonella sp. N(%)	P- value
Ampicillin (n=300)	R(n=289)	115(38)	2(0.6)	81(27)	85(28)	6(2)	0.000
	S(n=11)	5(2)	1(0.3)	0	0	5(1.6)	
Amox-clav (n=299)	R(n=199)	80(27)	1(0.3)	52(17)	66(22)	0	0.000
	S(n=100)	40(13)	2(0.6)	29(10)	19(6)	10(3)	
Piperacillin- tazobactam (n=301)	R(n=60)	34(11)	0	16(5)	10(3)	0	0.015
	S(n=241)	86(29)	3(0.9)	66(22)	75(25)	11(4)	
Ceftriaxone (n=300)	R(n=185)	95(32)	1(0.9)	44(15)	45(15)	0	0.000
	S(n=115)	25(8)	2(0.6)	37(12)	40(13)	11(4)	
Meropenem (n=293)	R(n=50)	16(5)	0	15(5)	19(6)	0	0.215
	S(n=243)	99(34)	3(1)	66(22)	64(22)	11(3)	
Amikacin (n=294)	R(n=69)	19(6)	1(0.3)	21(7)	28(9)	0	0.018
	S(n=225)	97(33)	2(0.6)	60(20)	55(18)	11(3)	
Ciprofloxacin (n=297)	R(n=135)	87(29)	0	22(7)	18(6)	8(2)	0.000
	S(n=162)	33(11)	3(1)	57(19)	66(22)	3(1)	
Fosfomycin (n=300)	R(n=94)	13(4)	1(0.3)	13(4)	67(22)	0	0.000
	S(n=206)	107(36)	2(0.6)	68(23)	18(6)	11(4)	
Neomycin (n=14)	R(n=4)	0	0	1(7)	3(21)	0	0.435
	S(n=10)	2(14)	1(7)	4(29)	3(21)	0	
Colistin (n=293)	R(n=13)	2(0.6)	3(1)	1(0.3)	7(2)	0	0.000
	S(n=280)	113(39)	0	79(27)	77(26)	11(3)	

R=resistance, S= sensitivity.

## DISCUSSION

Febrile neutropenia is frequently encountered by the institutes treating hematological disorders and is associated with increased morbidity and mortality. In our study, 403 bacterial isolates were collected from 242 patients suffering from various hematological disorders. The most prevalent organisms were gram negative bacteria which consisted of 74.9% (n=302) isolates however 25% (n=101) isolates revealed growth of gram positive organisms, as reported in previous studies.^13^ In the last two decades because of the use of quinolone prophylaxis that suppress the growth of gram negative bacilli and H2-receptor blockers which change gastric pH and promotes the growth of gram positive organisms, a changing pattern from gram negative to gram positive organisms is being reported in studies from developed countries.^14^ In developing countries, however the gram negative organisms are still more prevalent.^15^,^16^ Of the 403 isolates, maximum isolates were obtained from urine and peripheral blood cultures, as 39% and 21% respectively. The *S. aureus* comprised of 55% of gram positive isolates and it revealed a high resistance to penicillin but was sensitive to vancomycin. Methicillin resistant *S. aureus* (MRSA) prevalence in Pakistan is variable among different centers. Butt et al reported 40% prevalence of MRSA and in the same year Khan et al reported it 8%.^17^,^18^ Khawaja et al conducted study on patients of febrile neutropenia during two different periods and found 33% strains of MRSA during the both.^19^ In contrast, our study revealed 53% of MRSA isolates resistant to penicillin. The second most common gram-positive organism isolated in our study was *Enterococcus* sp. (36%) and around 39% of isolated *Enterococci* showed resistance to vancomycin. Vancomycin resistant *Enterococcus*(VRE) infections are usually hospital acquired infections and their spread have risen slowly.^20^

It has been reported that around 20-30% of nosocomial infections in United States are caused by *Enterococcu*s and labelled as the second most common cause of such infections worldwide.^21^ In our center we usually do not isolate VRE patients and this could be the cause spread of VRE and it should be minimized by strict isolation techniques such as use of gloves and gown before entering room and its removal before exit.^22^

Amongst the gram-negative organisms, *E. coli* turned out to be the most frequently isolated organism in our study followed by *P. aeruginosa* and *K. pneumoniae*. Local literature from Pakistan has also revealed *E.coli* to be the most frequently isolated gram negative organism.^10^,^23^ Around 38.3% of the *E.coli* isolates in present study exhibited higher resistance to penicillin and the similar results are reported by the other studies.^2^ Moreover, 39% of the *E. coli* isolates revealed high sensitivity to colistin.. Overall, piperacillin - tazobactam and meropenem resistance in gram negative organisms was found to be 20% and 17% respectively, while in gram positive organisms a comparatively higher rate of resistance i.e. 71% and 69% were seen.

Rising trend of carbapenem resistance against gram negative organisms has been reported in many studies worldwide.^3^,^10^ Contrasting findings were found in our study where resistance among gram-negative organisms against carbapenem and piperacillin/tazobactam was 20% and 17% and in gram positive organisms it was found to be 69% and 71% respectively.

Empirical antibiotic therapy currently used at our institute in febrile neutropenia is piperacillin/tazobactam along with amikacin. As infections with gram negative organisms are more prevalent in our setting, these bugs have shown considerable sensitivity to 1^st^ line empirical therapy. Among gram positive organisms *S. aureus* was seen as the most commonly isolated bug and majority of the isolates showed resistance to methicillin which is indeed a concern that needs attention. This resistance could be hospital environmental related or poor-quality control measures, which needs to be addressed and requires prompt review of institutional policies.

One of the possible reasons for antibiotics resistant bugs at our center could be the prophylactic use of fluoroquinolone which is given for seven days in chemotherapy induced neutropenia but it is increasingly associated with multidrug resistance.^24^ There is no evidence that suggests improved survival with the use of fluoroquinolone prophylaxis, so its use should be limited or discouraged.

Another reason behind the antibiotic resistance could be the overuse, inappropriate combination and inappropriate dosage of antibiotics during management of inpatient as well as outpatients. In our center, we routinely practice taking throat swabs for culture in almost every neutropenic patient with fever and treat the isolated organism with antibiotics which is not recommended, except in cases with suspected influenza like illness for detection of influenza or other viruses.^24^ This over treatment of throat isolated bugs could be another reason for undue exposure and antibiotic resistance.

Globally gram negative resistance to carbapenem is a matter of concern but in our data gram negative organisms are sensitive to carbapenem, however, the gram positive organisms are more resistant. The present data also highlighted that the *S. aureus* was highly resistant to methicillin, whereas *Enterococcus* *species* were resistant to vancomycin. By designing antibiotic regimens in accordance with the current data not only the overall survival of patients can be improved but the duration and cost of hospitalization can also be reduced. Furthermore, modalities like restricting harmful environmental exposure like liberal hospital visits of multiple attendants, hospital and personnel hygiene, education of nursing staff and patient’s attendants regarding hand hygiene, cough etiquette and proper handling/vaccination administration are some important aspects of patient care which should also be given importance because these factors do play a vital role in prevention and spread of infection with resistant bugs.^25^

### Author’s Contribution:

**UM, NA, NF, JH and TS:** had substantial contributions to the conception or design of the study, data collection, analysis and interpretation, and in manuscript writing. All authors did the revision of manuscript critically and approved the final version.

All authors are able to take public responsibility for the work and are agreed to be accountable for all aspects of the work in ensuring that questions related to the accuracy or integrity of any part of the work are appropriately investigated and resolved.
